# Blastic plasmacytoid dendritic cell neoplasm (BPDCN) arising in the setting of polycythemia vera (PV): An illustration of the emerging role of flow cytometry analysis in monitoring progression of myeloproliferative neoplasms

**DOI:** 10.1002/jha2.525

**Published:** 2022-07-03

**Authors:** Siba El Hussein, Mariko Yabe, Wei Wang, Naveen Pemmaraju, Sanam Loghavi, Fatima Zahra Jelloul, Hong Fang, L. Jeffrey Medeiros, W. Richard Burack, Andrew G. Evans, Jane L. Liesveld, John M. Bennett

**Affiliations:** ^1^ Department of Pathology University of Rochester Medical Center Rochester New York United States; ^2^ Hematopathology Service Department of Pathology and Laboratory Medicine Memorial Sloan Kettering Cancer Center New York New York United States; ^3^ Department of Hematopathology The University of Texas MD Anderson Cancer Center Houston Texas United States; ^4^ Department of Leukemia The University of Texas MD Anderson Cancer Center Houston Texas United States; ^5^ Department of Medicine & The Wilmot Cancer Institute University of Rochester Medical Center Rochester New York United States

**Keywords:** blastic plasmacytoid dendritic cell neoplasm, myeloproliferative neoplasm, polycythemia vera

## Abstract

This report highlights the value of flow cytometry analysis, particularly in the setting of myeloproliferative neoplasms showing features of progression, as neoplastic plasmacytoid dendritic cell (PDC) proliferations may be present, representing either a clonal expansion of mature PDCs related to the underlying myeloproliferative neoplasm or transformation to blastic plasmacytoid dendritic cell neoplasm (BPDCN). BPDCN should always be considered in patients with myeloid neoplasms in progression and/or who develop new cutaneous findings, as it may prompt change of management.

## 1 INTRODUCTION

**FIGURE 1:**
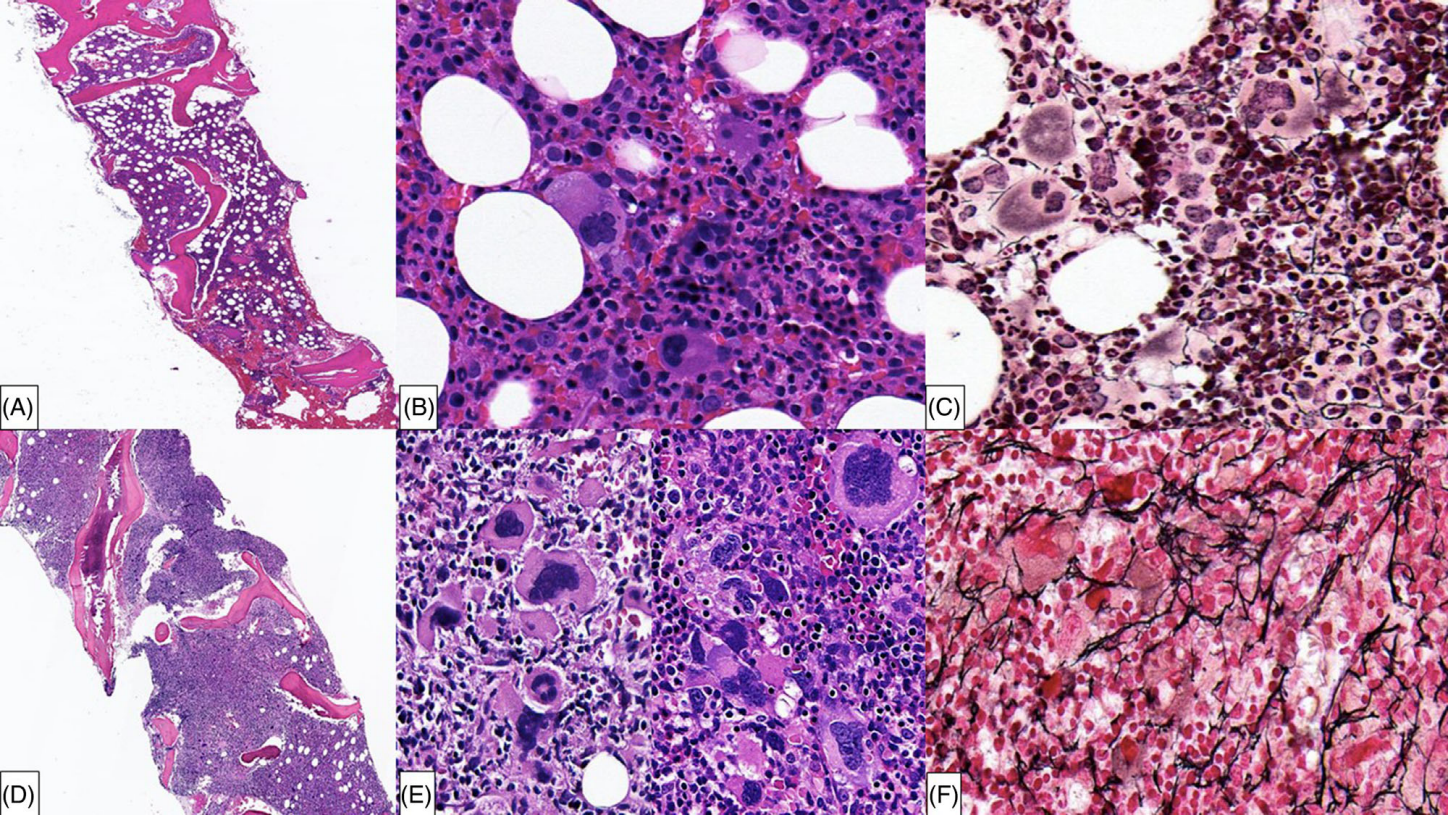
Two bone marrow biopsy specimens demonstrating morphologic evolution of patient's polycytemia vera: (A) Initial bone marrow biopsy with increased cellularity and (B) panmyelosis (C) without significant reticulin fibrosis. Subsequent bone marrow biopsy during disease acceleration (D) with increased marrow cellularity, (E) frequent clusters of megakaryocytes, and (F) increased background reticulin fibrosis (MF2)

**FIGURE 2:**
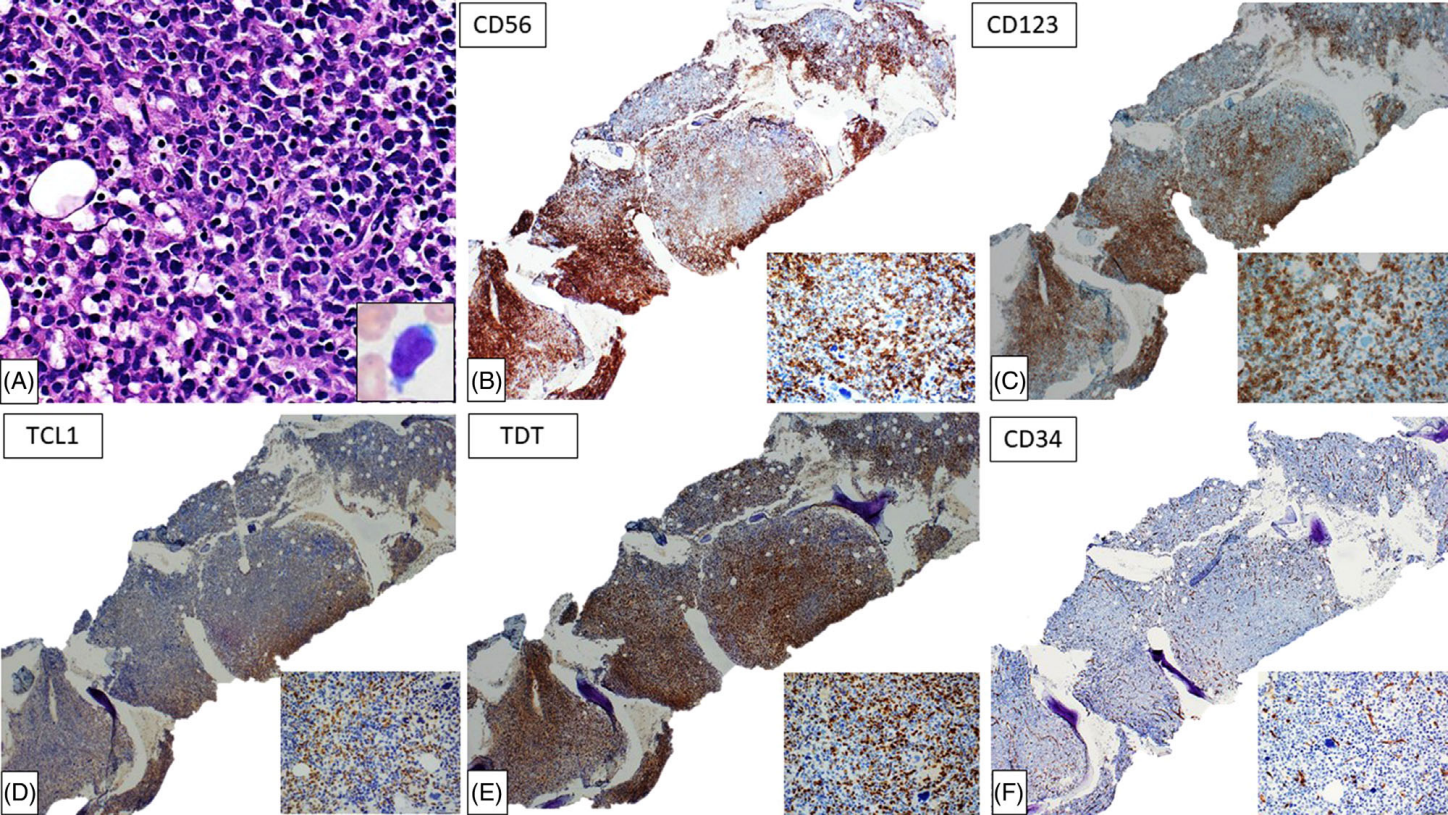
Immunohistochemical characterization of the blastic plasmacytoid dendritic cell neoplasm (BPDCN) population in the bone marrow: (A) High power view illustrating a focus of blastic plasmacytoid dendritic cells in the marrow core biopsy, inset illustrating one blastic plasmacytoid dendritic cell in marrow aspirate smear. The neoplastic cells in the marrow core biopsy are positive for (B) CD56, (C) CD123, (D) TCL1, and (E) TDT expression, while negative for (F) CD34 expression by immunohistochemistry

An 80‐year‐old man, diagnosed with a *JAK2* V617F mutation positive‐polycythemia vera (PV) (Figure 1A–C) with a diploid karyotype in 2011, treated with hydroxyurea and phlebotomies, presented in 2021 with increasing fatigue and unintentional weight loss. Physical exam showed splenomegaly measuring 20.5 cm on computerized tomography (CT) scan. Complete blood count showed pancytopenia: White blood cells 1.2 K/μL, hemoglobin 11.4 g/dL, mean corpuscular volume 84.6 fL, and platelets 63K/μL. No circulating blasts were seen. Bone marrow biopsy revealed a hypercellular marrow (> 90%) (Figure 1D) with atypical megakaryocytic hyperplasia (Figure 1E). Granulocytes showed progressive maturation and erythroid cells showed left‐shifted maturation. Reticulin stain demonstrated grade 2 myelofibrosis (MF‐2) (Figure 1F). This constellation of findings along with the clinical presentation was consistent with the diagnosis of post‐PV myelofibrosis. Flow cytometry analysis on the concurrent marrow aspirate showed no increased myeloblasts. Instead, aberrant plasmacytoid dendritic cells (PDC) (13% of total analyzed cells) were detected, expressing CD2, CD7 (partial), CD11c, CD33, CD38, CD45 (dim), CD56, CD103, CD123 (bright), and HLA‐DR. This PDC population was negative for CD3, CD4, CD5, CD8, CD14, CD64, CD117, and cytoplasmic MPO expression (Figure S1A–F). In light of the flow cytometry immunophenotypic results, a battery of immunostains was performed on the bone marrow core biopsy to confirm the presence of PDCs: consistent with flow cytometry, clusters of PDCs were identified (∼30% of marrow cellularity) (Figure 2A), expressing CD56, CD123, TCL1, and TDT (Figure 2B–E), while negative for CD34 (Figure 2F) and CD117 expression. Myeloblasts were not increased by morphologic and immunophenotypic (flow cytometry and immunohistochemical) analysis. Few PDCs were also morphologically identified on marrow aspirate smears (Figure 2A, *inset*). Conventional cytogenetic analysis revealed the following karyotype: 46, XY, del (20) (q11.2q13) [19]/46, XY [1]. Next‐generation sequencing analysis was not performed. The diagnosis of blastic plasmacytoid dendritic cell neoplasm (BPDCN) was rendered on the bone marrow biopsy, which prompted the search for potential skin involvement by BPDCN. Erythematous papular lesions were identified on the anterior chest and were biopsied (Figure S2A). Microscopic examination revealed a superficial and deep dermal skin infiltrate (Figure S2B) composed of neoplastic PDCs (Figure S2C), expressing CD56 and CD123 (Figure S2D–E), consistent with the diagnosis of BPDCN. CD4 expression was negative in the neoplastic cells and positive in background admixed histiocytes (Figure S2F). Next‐generation sequencing interrogating 35 unique genes including *JAK2* (Table S1) with a limit of detection at 5% variant allele frequency at 500× coverage, was negative. Cerebrospinal fluid was negative for involvement by BPDCN. The patient was started on tagraxofusp, an anti‐CD123 antibody conjugated to a truncated diphtheria toxin fusion protein. The skin lesions disappeared rapidly and blood counts normalized after cycle one. He tolerated two cycles of tagraxofusp well. However, the third cycle was interrupted due to insomnia, anxiety, and fever. Magnetic resonance imaging and lumbar puncture analysis did not show central nervous system involvement; nevertheless, the patient passed away four weeks after.

## 2 DISCUSSION

PDCs are a subtype of dendritic cells with the function of interferon production and antigen presentation. Expansion of PDCs with an associated myeloid neoplasm is occasionally encountered in clinical practice through two main scenarios, each individual scenario harboring its own clinical and diagnostic complexities: (1) myeloid neoplasms with PDC expansion, without underlying BPDCN (“fully mature”, as it has been documented in cases of CMML [1, 2, 3, 4], or “undergoing maturation” as it has been recently described in non‐CMML myeloid neoplasms [5, 6, 7, 8, 9]) and (2) coexisting myeloid neoplasms and BPDCN [10, 11] (clonally related or unrelated). The current report illustrates the second scenario, in which a patient with previous history of PV presented in accelerated phase, with concomitant BPDCN, involving the skin and bone marrow. In addition to PDC expansion, there are cases of acute myeloid leukemia with PDC‐like phenotype, without underlying BPDCN [12, 13, 14], in which myeloblasts show immunophentypic overlap with PDCs [6, 15].

Similar to systemic mastocytosis with associated hematologic malignancies (SM‐AHM), 10%–20% of BPDCN cases are associated with another hematologic neoplasm that may precede, coexist with, or follow BPDCN. “BPDCN with prior and/or concomitant hematologic malignancies” (BPDCN‐PCHM) is most often reported with MDS and CMML, and rarely multiple myeloma [11]. However, plausible theories for the etiology of BPDCN‐PHCM include the divergent differentiation of a common stem cell, or progression from underlying myeloid neoplasm [6]. The two neoplastic processes in the current case (PV and BPDCN) may be (1) clonally related as described in AML with BPDCN, supporting the theory of evolution of BPDCN from underlying myeloid neoplasms, (2) divergent yet possibly stemming from a common hematopoietic stem cell (possibly the scenario in the presented case, as *JAK2* mutation was not detected in the BPDCN involving the skin), or (3) completely unrelated. Whether the marrow BPDCN component played a role in PV progression at the microenvironment level remains unclear. Nevertheless, this case highlights the value of flow cytometry analysis in characterizing aberrant populations (blastic PDCs in this case), otherwise not expected in the setting of an established disease, especially since foci of BPDCN in bone marrow biopsies could be missed by morphologic examination alone, and more so in the setting of a hypercellular marrow involved by an underlying myeloproliferative neoplasm in progression. Furthermore, negative CD4 expression encountered in a minor subset of BPDCN was encountered in the current case (Figure S1C and 2F), making the diagnosis even more challenging in cases without a previously established BPDCN. Thus, in similar scenarios, applying a complete battery of PDC‐related markers [11] is recommended to make an accurate diagnosis of BPDCN.

## 3 CONCLUSION

In summary, we describe a case of CD4‐negative BPDCN coexisting with progressive PV, first suggested by flow cytometry analysis of the marrow, then identified involving the skin. This case highlights the value of flow cytometry analysis particularly in the setting of myeloproliferative neoplasms showing features of progression, as distinct PDC proliferations may be present, either representing a clonal expansion related to the underlying myeloproliferative neoplasm without underlying BPDCN [6, [8], or portraying the leukemic counterpart of an as yet undiagnosed underlying BPDCN. This case is a reminder that BPDCN should always be considered in patients with myeloid neoplasms in progression and/or who develop new cutaneous findings as it may prompt the care team to change management. This could include addition of anti‐CD123, different cytotoxic chemotherapy agent, central nervous system‐directed therapy, hypomethylating agents with BCL2‐antagonists, or inclusion of consideration for stem cell transplantation in appropriate cases.

## FUNDING

The authors received no specific funding for this work.

## CONFLICT OF INTEREST

The authors declare they have no conflicts of interest.

## ETHICS APPROVAL STATEMENT

The patient signed the institution‐approved standard consent for clinical diagnostic testing.

## Supporting information

FIGURE S1. Flow cytometric characterization of the blastic plasmacytoid dendritic cell neoplasm population in the bone marrow: (A) Flow cytometry analysis of bone marrow aspirate reveals an aberrant population in the CD45 dim gate (highlighted in blue), representing ∼13% of total analyzed cells, negative for (B) CD34, CD117, (C) CD3 and CD4 expression, while positive for (D) CD7, (E) CD2, CD56, (F) HLA‐DR and CD123 (bright) expression

FIGURE S2. Blastic plasmacytoid dendritic cell neoplasm involving the skin: (A) Erythemtous papular lesions involving the skin of the chest; (B) histologic examination of a punch biopsy of the skin lesions reveals a dermal infiltrate (C) composed of neoplastic plasmacytoid dendritic cells, expressing (D) CD56 and (E) CD123, while negative for (F) CD4 expression

TABLE S1. Next‐generation sequencing panel
