# Supplementary figures and images for: Blastic plasmacytoid dendritic cell neoplasm (BPDCN) arising in the setting of polycythemia vera (PV): An illustration of the emerging role of flow cytometry analysis in monitoring progression of myeloproliferative neoplasms
Source: EJHaem. 2022 Jul 3;3(3):954–7. doi: 10.1002/jha2.525 (PMC9421993; doi:10.1002/jha2.525)

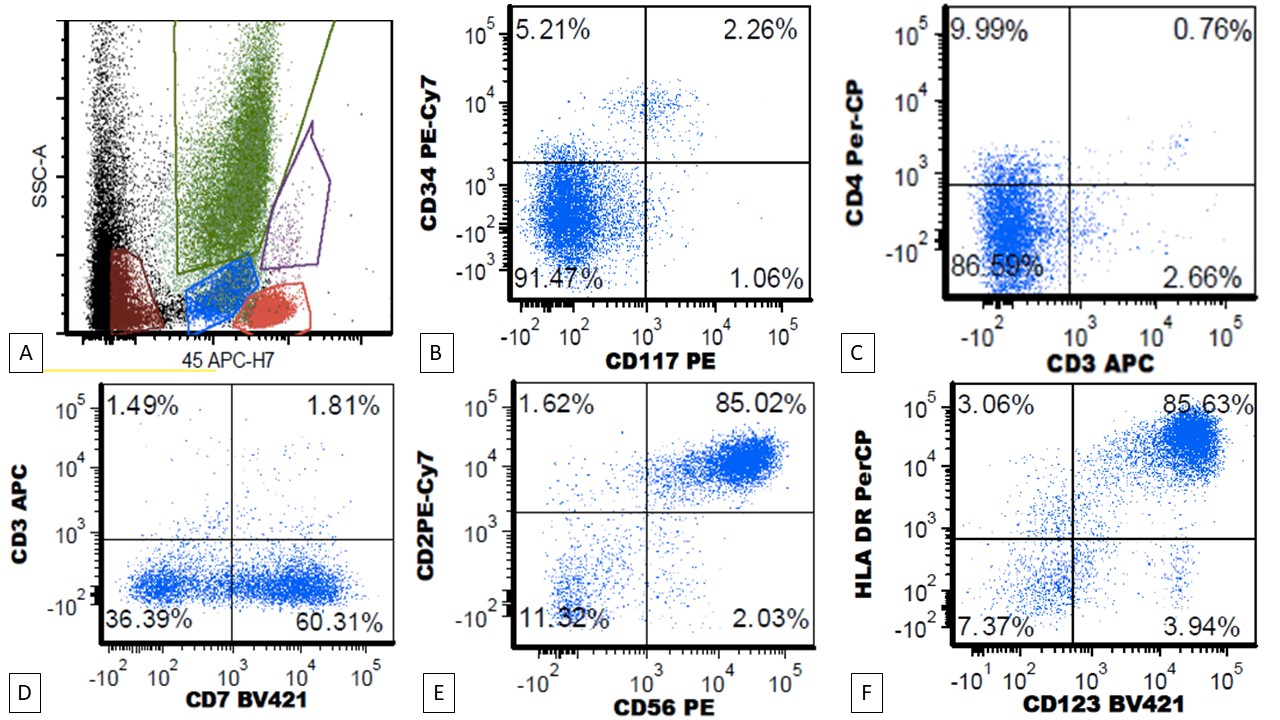

Supplement: Supplementary file 1 — FIGURE S1. Flow cytometric characterization of the blastic plasmacytoid dendritic cell neoplasm population in the bone marrow: (A) Flow cytometry analysis of bone marrow aspirate reveals an aberrant population in the CD45 dim gate (highlighted in blue), representing ∼13% of total analyzed cells, negative for (B) CD34, CD117, (C) CD3 and CD4 expression, while positive for (D) CD7, (E) CD2, CD56, (F) HLA‐DR and CD123 (bright) expression [file JHA2-3-954-s003.JPG]

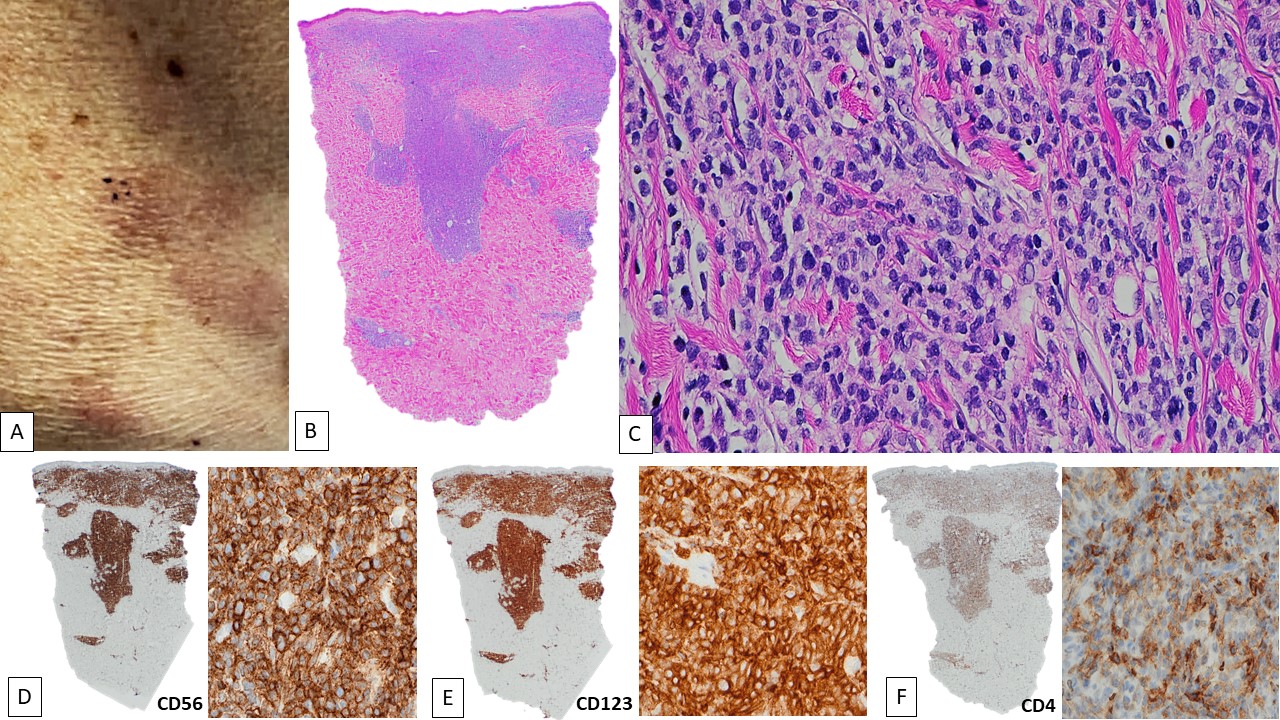

Supplement: Supplementary file 2 — FIGURE S2. Blastic plasmacytoid dendritic cell neoplasm involving the skin: (A) Erythemtous papular lesions involving the skin of the chest; (B) histologic examination of a punch biopsy of the skin lesions reveals a dermal infiltrate (C) composed of neoplastic plasmacytoid dendritic cells, expressing (D) CD56 and (E) CD123, while negative for (F) CD4 expression [file JHA2-3-954-s002.JPG]
