# Supplementary material for: Blastic plasmacytoid dendritic cell neoplasm (BPDCN) arising in the setting of polycythemia vera (PV): An illustration of the emerging role of flow cytometry analysis in monitoring progression of myeloproliferative neoplasms
Source: EJHaem. 2022 Jul 3;3(3):954–7. doi: 10.1002/jha2.525 (PMC9421993; doi:10.1002/jha2.525)
Supplement: Supplementary file 3 — TABLE S1. Next‐generation sequencing panel [file JHA2-3-954-s001.docx]

| AKT1 | ALK | AR | BRAF | CDK4 | CTNNB1 | DDR2 |
| --- | --- | --- | --- | --- | --- | --- |
| EGFR | ERBB2 | ERBB3 | ERBB4 | ESR1 | FGFR2 | FGFR3 |
| GNA11 | GNAQ | HRAS | IDH1 | IDH2 | JAK1 | JAK2 |
| JAK3 | KIT | KRAS | MAP2K1 | MAP2K2 | MET | MTOR |
| NRAS | PDGFRA | PIK3CA | RAF1 | RET | ROS1 | SMO |

**Supplemental 1.** **Next generation sequencing panel**
